# Corrosion Behavior and Degradation Mechanism of High-Alloy Bainitic Gray Cast Iron Cylinder Liners for Methanol Engines in Formic Acid–NaCl Media

**DOI:** 10.3390/ma19142950

**Published:** 2026-07-09

**Authors:** Song-Bo Yang, Wen-Juan Zhang, Hao Gao, Ya-Hui Xue, Qi-Fei Hou, Dong Liu, Hai-Tao Wang, Guo-Zheng Quan

**Affiliations:** 1 School of Mechanical and Electric Engineering, Sanming University, Sanming 365004, China; 2 ZYNP International Corporation, Industrial Cluster District, Mengzhou 454750, China; 3 Henan Key Laboratory of Advanced Internal Combustion Engines Friction Pair and Lubrication, Mengzhou 454750, China; 4 Chongqing Key Laboratory of Advanced Mold Intelligent Manufacturing, School of Material Science and Engineering, Chongqing University, Chongqing 400044, China

**Keywords:** methanol-fueled engine, high-alloy bainitic gray cast iron, formic acid corrosion, QPQ treatment, nitriding–oxidizing treatment, microgalvanic corrosion

## Abstract

Methanol is a promising alternative fuel for internal combustion engines; however, formic acid (methanoic acid, HCOOH) formed or enriched during engine operation can corrode cylinder liners and reduce cylinder liner–piston ring reliability. In this study, high-alloy bainitic gray cast iron cylinder liners were investigated in formic acid–NaCl media under three surface states: untreated substrate, quench–polish–quench (QPQ) treatment, and nitriding–oxidizing treatment. Static full-immersion tests were conducted in 5 and 10 vol% formic acid–NaCl solutions, and corrosion damage was evaluated by mass loss, mass-loss-equivalent average corrosion depth, and cross-sectional SEM-BSE observation. With increasing solution aggressiveness, all specimens showed increased corrosion depth and mass loss. For the untreated substrate, these values increased from 97.65 to 146.05 μm and from 929.8 to 1391.2 mg, respectively; the corresponding changes were 61.45–112.55 μm and 545.0–997.3 mg for QPQ-treated specimens and 55.25–119.00 μm and 488.7–1054.6 mg for nitriding–oxidizing-treated specimens. Under the lower-severity condition, nitriding–oxidizing and QPQ treatments reduced the mass-loss-equivalent corrosion depth by 43.42% and 37.07%, respectively. Cross-sectional observations indicate that flake graphite/bainitic-matrix microgalvanic coupling, formate-assisted dissolution, local degradation of modified surface regions, and defect-assisted electrolyte penetration jointly promoted inward corrosion. These results provide guidance for corrosion-resistant surface design of methanol-engine cylinder liners.

## 1. Introduction

The global transition toward low-carbon energy systems and the implementation of the dual carbon strategy in China have accelerated the search for clean liquid fuels for internal combustion engines. Methanol has attracted considerable attention because it can be produced from coal, biomass, renewable hydrogen, and captured CO_2_ and remains liquid under typical room-temperature conditions (approximately 20–25 °C), which facilitates storage, distribution, and engine application [1,2,3,4,5,6]. Recent reviews and life-cycle assessments have demonstrated that methanol vehicles and methanol-fueled heavy-duty or marine engines can reduce petroleum dependence and improve emission performance, especially when green methanol production routes are adopted [2,3,7,8,9,10]. However, the wider use of methanol engines depends not only on fuel economy and emissions performance but also on material corrosion reliability [11,12,13,14]. During methanol combustion, blow-by condensation, and low-temperature operation, oxygenated products such as formaldehyde and formic acid may be generated or enriched. When formic acid comes into contact with condensed water and chloride ions, the resulting environment is highly aggressive toward iron-based components, promoting acid dissolution, passive-film degradation, and localized corrosion [12,15,16,17,18,19,20,21,22]. Clarifying the corrosion behavior of cylinder liners in formic acid media is essential for reliable methanol-engine application because cylinder liners directly affect sealing stability, heat transfer, and service life.

Recent research on alternative liquid fuels has increasingly recognized corrosion as a limiting factor in large-scale engineering applications [11]. Regarding methanol-related combustion products, Cai et al. investigated cast iron cylinder liners in simulated ammonia-, methanol-, and diesel-combustion-product solutions and found that the methanol-related medium produced more severe corrosion, with flake graphite in cast iron promoting preferential dissolution of the surrounding metallic matrix [12]. Recent work on methanol-engine core friction pairs and alcohol-fuel-blend corrosion further indicates that acidic combustion products such as methanoic acid and formaldehyde can aggravate corrosion and wear of gray-cast-iron cylinder liners, while alcohol-containing fuels may promote electrochemical corrosion of steels when water, oxygenated species, and other contaminants are present [13,14]. Weak organic acid corrosion has also been systematically investigated in steel and stainless steel systems. Formic acid can accelerate corrosion through dissociation-driven H^+^ supply, local pH buffering, and Fe^2+^ complexation, whereas high formic acid concentrations may induce a passivation-to-activation transition [15,16,17,18,19,20]. The protective capability of magnetite can also be weakened by HCOOH, indicating that Fe_3_O_4_-containing surface films may not remain stable in formic acid–NaCl environments [17]. In addition, chloride ions can penetrate passive films, generate defect channels, and induce localized film breakdown, making the combined action of H^+^, HCOO^−^, and Cl^−^ more destructive than that of a neutral NaCl medium alone [21,22]. Recent experimental and simulation studies on iron and steel corrosion have further emphasized that microscale surface chemistry, water/oxygen adsorption, and local electrochemical heterogeneity strongly influence the initiation and propagation of corrosion [23,24,25].

Although these studies have provided a strong foundation, several issues remain unresolved in the present material system. First, most corrosion studies related to methanol engines have focused on conventional gray cast iron, general steels, or stainless steels, whereas the corrosion evolution of high-alloy bainitic gray cast iron cylinder liners in formic acid–NaCl solutions remains insufficiently understood. This material consists of a bainitic metallic matrix and interconnected flake graphite. Although graphite contributes to the typical service characteristics of gray cast iron, it also introduces electrochemical heterogeneity in acidic electrolytes. Once the graphite–matrix interface is exposed, graphite may act as a relatively cathodic phase, whereas the adjacent bainitic matrix is prone to preferential anodic dissolution. Recent studies on cast irons have shown that graphite morphology, matrix structure, alloying elements, corrosion products, and service environment significantly influence localized corrosion behavior and the formation or disruption of protective rust layers [26,27,28,29,30]. However, these findings cannot fully explain the penetration behavior of formic acid and chloride-containing media through the graphite network in high-alloy bainitic gray cast iron. In addition, macroscopic mass-loss measurements alone cannot reveal how corrosion initiates at local defects, penetrates modified surface regions, and propagates along graphite–bainite interfaces. Therefore, an integrated evaluation based on mass loss, mass-loss-equivalent average corrosion depth, and representative cross-sectional microscopic morphology is necessary.

Surface modification is a practical approach for improving the corrosion resistance of Fe-based engine materials [31]. Nitriding, nitrocarburizing, quench–polish–quench (QPQ) treatment, and nitriding followed by oxidation can produce compound layers, diffusion regions, and oxide films such as Fe_3_O_4_/Fe_2_O_3_, which may act as physical barriers and reduce direct contact between the substrate and the corrosive medium [32,33,34,35]. Nevertheless, many recent studies have evaluated these surface layers in neutral chloride solutions or general corrosion environments. Their protective performance and failure limits under the combined action of formic acid, formate ions, and chloride ions remain unclear, particularly for graphite-containing cast iron cylinder liners. This study investigated high-alloy bainitic gray cast iron cylinder liners in formic acid–NaCl mixed solutions to address these gaps. Three surface states were compared: untreated substrate, QPQ-treated samples, and nitriding–oxidizing-treated samples. Static full-immersion corrosion tests, mass-loss measurements, mass-loss-equivalent corrosion-depth calculations, cross-sectional morphology analyses, and selected EDS elemental mapping were conducted to reveal the coupled effects of acid-assisted dissolution, formate complexation, chloride-assisted localized attack, and graphite–bainite microgalvanic corrosion. The results provide an experimental basis for the low-cost, corrosion-resistant surface design of cylinder liners for methanol engines.

## 2. Materials and Methods

### 2.1. Materials

**Figure 1:**
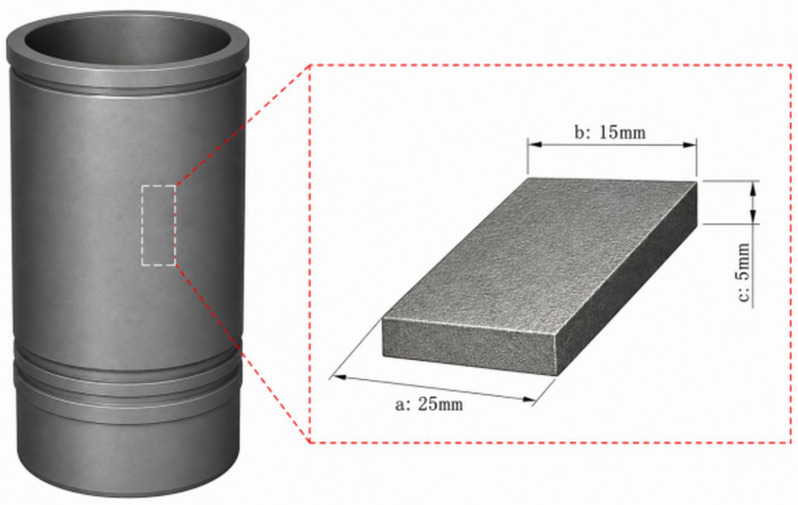
Schematic diagram of the sampling position and geometry of high-alloy bainitic gray cast iron cylinder liner corrosion specimens.

The substrate material used in this study was a commercial high-alloy bainitic gray cast iron cylinder liner supplied by ZYNP International Corporation, Mengzhou, China. The cylinder liners were produced from the same industrial batch by centrifugal casting followed by the producer’s bainitic heat-treatment process. Rectangular specimens were machined from the designated wall region of the cylinder liner by wire electrical discharge machining, as shown in Figure 1. Each specimen measured 25 mm × 15 mm × 5 mm.

Three surface states were investigated: untreated substrate, QPQ-treated surface, and gas nitriding–oxidizing-treated surface. The untreated substrate specimens were taken before any QPQ or gas nitriding–oxidizing treatment and were used as the reference material. In this study, “bainitic gray cast iron” refers to gray cast iron with a bainitic metallic matrix, rather than single-phase bainite or steel. The substrate microstructure mainly consists of an acicular bainitic matrix and flake graphite, with possible minor alloy-enriched carbides or secondary constituents. The chemical composition is listed in Table 1.

### 2.2. Surface Modification Treatments and Corrosion Tests

Two surface modification routes were applied to the high-alloy bainitic gray cast iron cylinder liners: quench–polish–quench (QPQ) treatment and gas nitriding–oxidizing treatment. The treated specimens were not mechanically polished after surface modification so that the modified surface regions could be preserved before corrosion testing.

For the QPQ treatment, the specimens were preheated at 400 °C for 1 h, salt-bath nitrocarburized at 540 °C for 3.5 h, oxidized at 390 °C for 20 min, and then water-cooled. This treatment was intended to form a nitrocarburized surface region and an oxidized outer surface. For the gas nitriding–oxidizing treatment, the specimens were preheated in an N_2_ atmosphere at 350 °C for 1 h, nitrided in an NH_3_ atmosphere at 540 °C for 4 h, steam-oxidized in an H_2_O atmosphere at 415 °C for 1 h, and then furnace-cooled. This treatment was intended to form an oxidized outer region on an N-enriched nitrided region. The detailed processing parameters are summarized in Table 2.

**Figure 2:**
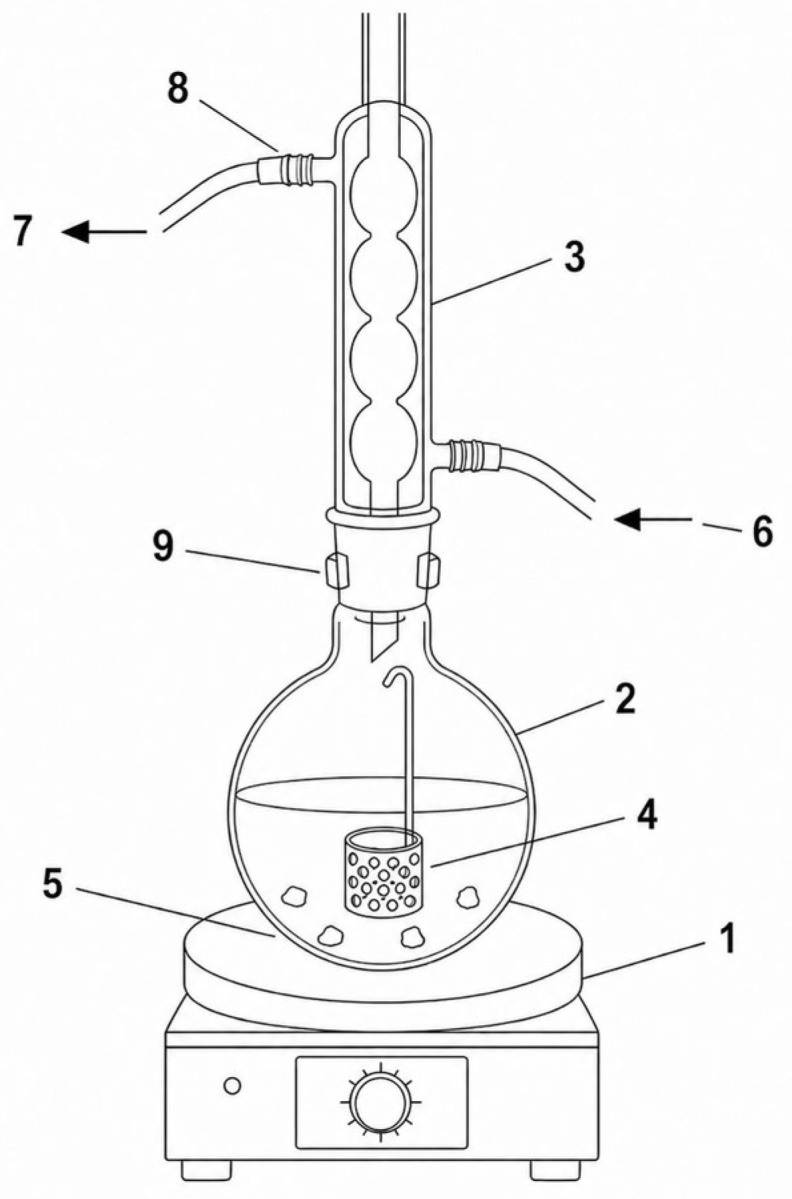
Schematic diagram of the static full-immersion corrosion-test apparatus: 1—heating device; 2—round-bottom flask; 3—Allihn bulb condenser; 4—glass specimen holder; 5—boiling chips; 6—water inlet; 7—water outlet; 8—metal wire fixing the condenser; 9—ground-glass joint coated with silicone oil.

Static full-immersion corrosion tests were conducted using the reflux apparatus shown in Figure 2. The corrosive solution was contained in a round-bottom flask placed on a heating device, and the specimen was suspended in the solution using a glass holder. An Allihn condenser was connected above the flask to reduce evaporation during heating. Cooling water entered from the lower port and exited from the upper port. Boiling chips were added to prevent bumping, and the ground-glass joint was sealed with silicone oil.

The corrosion tests followed the relevant procedures of JB/T 7901 [36] and GB/T 21621 [37]. Before exposure, the untreated substrate specimens were ground and polished to remove machining marks. The QPQ-treated and gas nitriding–oxidizing-treated specimens were not polished after treatment, so their modified surface regions remained intact. All specimens were ultrasonically cleaned sequentially in deionized water, absolute ethanol, and acetone; dried with hot air; and cooled in a desiccator before being weighed.

Two formic acid–NaCl media were used, as listed in Table 3. Solution #1 contained 50 mL formic acid and 2.5 g NaCl diluted to 1000 mL with deionized water and was tested at 60 °C. Solution #2 contained 100 mL formic acid and 2.5 g NaCl diluted to 1000 mL and was tested at 80 °C. The immersion time was 60 min for all conditions. Each specimen was immersed separately in a 500 mL borosilicate glass vessel to avoid unintended galvanic coupling or local shielding.

Solution #1 was selected as a lower-severity reference condition, whereas Solution #2 was designed as a more aggressive accelerated condition by increasing both the formic acid concentration and the test temperature. These two media were used to compare the relative corrosion resistance of the three surface states under two representative severity levels, rather than to establish a complete concentration-temperature matrix. The higher acid concentration was paired with a higher temperature to accelerate acid- and temperature-assisted degradation of the modified surface regions within a short immersion time.

Before immersion, the length, width, and thickness of each specimen were measured to calculate the exposed surface area, *S*, and the initial mass, *M*_1_, was recorded. After exposure, the specimen was removed, ultrasonically cleaned in absolute ethanol to remove loose corrosion products and residual acid, dried with hot air, kept in a desiccator for 30 min, and weighed again to obtain the final mass, *M*_2_. Two parallel specimens were tested for each surface state and solution condition, and the results are reported as mean values with standard deviations.

The annualized corrosion-rate index, *v*, calculated from mass loss, was used only as a comparative index under accelerated test conditions:

(1) $$v\;=\;K\;\times \;(M_{1}\;-\;M_{2})/(S\;\times \;t\;\times \;\rho )\;$$

where *K* is the unit-conversion coefficient, *ρ* is the material density, *S* is the exposed surface area, and *t* is the immersion time. In this study, *K* was 8760 h year^−1^. The density, *ρ*, was taken as the density of the same high-alloy bainitic gray cast iron cylinder liner material, and the same value was used for all specimens because they were machined from the same material batch. The mass-loss-equivalent average corrosion depth was calculated by dividing the volume loss by the exposed surface area. This parameter was used for overall quantitative comparison, while cross-sectional SEM-BSE observation was used to evaluate representative local corrosion-front morphology.

### 2.3. Characterization

The untreated substrate was characterized by SEM-BSE observation before corrosion testing to document the graphite-containing bainitic gray cast iron microstructure. The uncorroded QPQ-treated and gas nitriding–oxidizing-treated samples were characterized by SEM-BSE observation and EDS elemental mapping of selected elements to evaluate the modified surface regions. C, N, O, and Fe were used to describe graphite distribution, nitrogen-enriched regions, oxidation features, and the metallic matrix, respectively. After corrosion testing, the specimens were sectioned along the longitudinal direction, cold-mounted in epoxy resin, ground, polished stepwise, and examined by cross-sectional SEM-BSE observation. Local corrosion-affected widths were measured at selected positions in the cross-sections to describe corrosion-front morphology and penetration paths. For the corroded cross-sections, selected EDS elemental mapping of C, O, and Fe was performed to correlate elemental distributions with the marked SEM-BSE morphological regions in representative corrosion-affected areas.

In addition to qualitative elemental-distribution comparison, the O Kα EDS maps of the corroded specimens were further processed using a semi-quantitative image-based method. A consistent RGB-thresholding criterion was applied to all selected O maps. The black background outside the effective mapping region and image labels were excluded. The calculated O-enriched area fraction was used only to compare relative oxygen-enriched regions among specimens, rather than to represent absolute oxygen concentration in wt% or at.%.

The immersion tests were performed using a constant-temperature water bath (HH-4; Shanghai Shuangxu Electronics Co., Ltd., Shanghai, China). Specimen masses before and after corrosion were measured using an electronic balance (MS105DU; Mettler Toledo MTCN Limited Company, Hong Kong, China; accuracy 0.1 mg). Microstructural and cross-sectional observations were conducted using a tungsten-filament scanning electron microscope (EVO18; Zeiss, Jena, Germany) equipped with a backscattered electron detector and operated in BSE mode. Elemental mapping was performed using an EDS system (X-MAX; Oxford Instruments, Oxford, UK). Surface profile and local corrosion-depth measurements were supported by a roughness/profile measuring instrument (T8000; Hommelwerke, Villingen-Schwenningen, Germany), and local corrosion-affected widths and selected cross-sectional morphological features were measured using an image-analysis system (293; Best Instrument Co., Ltd., Suzhou, China). The O Kα EDS maps were processed using Python software (version 3.13.5; Python Software Foundation, Wilmington, DE, USA), together with OpenCV (version 4.13.0) and NumPy (version 2.3.5), for RGB thresholding and oxygen-enriched area-fraction calculation.

## 3. Results and Discussion

### 3.1. Characterization of the Substrate and Surface-Modified Regions Before Corrosion

**Figure 3:**
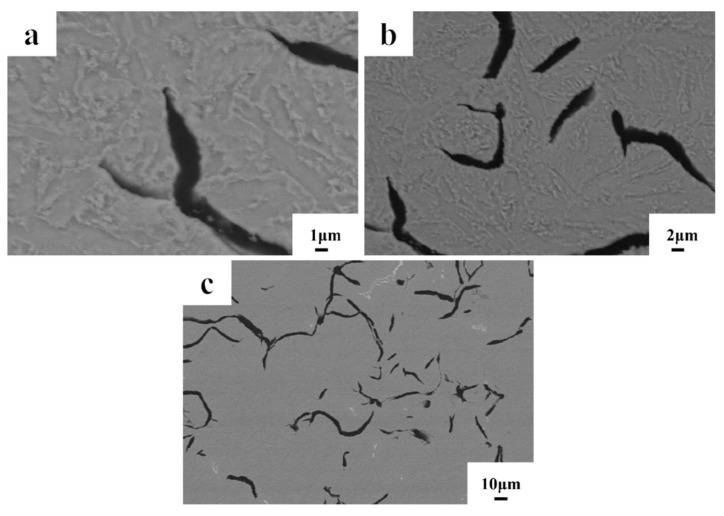
SEM-BSE microstructures of the untreated high-alloy bainitic gray cast iron substrate before QPQ or gas nitriding–oxidizing surface treatment: (**a**) high-magnification image showing the acicular bainitic matrix and flake graphite; (**b**) medium-magnification image showing the graphite/matrix interface; and (**c**) low-magnification image showing the distribution of flake graphite in the substrate.

Before corrosion testing, the untreated substrate and the two surface-modified samples were characterized to establish the initial microstructural state of the cylinder liner material. Figure 3 shows the SEM-BSE microstructures of the untreated high-alloy bainitic gray cast iron substrate before QPQ or gas nitriding–oxidizing treatment. The specimen shown in Figure 3 was the untreated cast iron substrate rather than steel and was not subjected to additional surface modification; it was ground, polished, etched, and examined by SEM-BSE observation.

The untreated substrate exhibits a graphite-containing bainitic gray cast iron structure rather than a single-phase bainite structure. Dark flake graphite is distributed within the acicular bainitic matrix, and the graphite/matrix interfaces are clearly visible. The bainitic matrix provides the continuous metallic framework of the cylinder liner, whereas flake graphite forms the characteristic discontinuous carbon phase of gray cast iron. This microstructure introduces local interfacial heterogeneity and provides preferential sites for electrolyte retention and localized attack during subsequent exposure.

**Figure 4:**
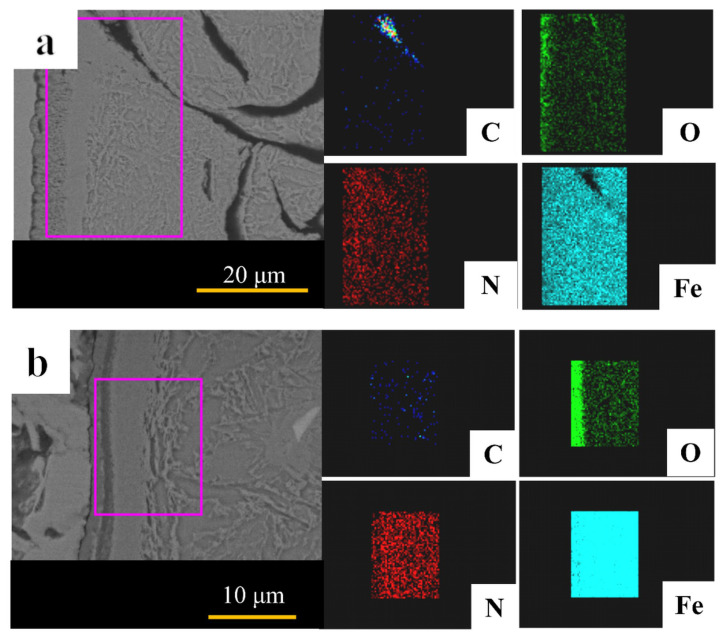
SEM-BSE images and selected EDS elemental mapping results of the uncorroded surface-modified regions: (**a**) QPQ-treated sample at 5000× and (**b**) gas nitriding–oxidizing-treated sample at 8000×. The purple rectangles in the SEM-BSE images indicate the regions selected for EDS elemental mapping. In the EDS maps, blue, green, red, and cyan indicate the relative distributions of C, O, N, and Fe, respectively. The color intensity represents the relative elemental signal distribution in the selected regions. The yellow scale bars indicate 20 μm in (**a**) and 10 μm in (**b**).

The processing parameters of the QPQ and gas nitriding–oxidizing treatments are summarized in Table 2. To verify the formation and elemental features of the modified surface regions before corrosion, the uncorroded QPQ-treated and gas nitriding–oxidizing-treated samples were analyzed by SEM-BSE observation and EDS elemental mapping, as shown in Figure 4.

For the QPQ-treated sample, the SEM-BSE image shows a micrometer-scale modified region near the outer surface, and the purple rectangle marks the region selected for EDS elemental mapping. The N map shows a relatively enriched signal in the selected near-surface region, consistent with nitrogen introduction during salt-bath nitrocarburizing. O is detected close to the surface, which is consistent with the subsequent oxidation step. Fe is mainly distributed in the metallic matrix, and C-rich features are associated with flake graphite. These elemental features are consistent with the formation of a nitrocarburized/oxidized surface region before corrosion.

For the gas nitriding–oxidizing-treated sample, the SEM-BSE image and the marked EDS mapping region show a distinguishable near-surface modified region. The O map shows a relatively enriched band close to the outer surface, consistent with steam oxidation, while N is distributed in the modified region produced by NH_3_ gas nitriding. The Fe map identifies the metallic matrix, and the C map mainly corresponds to graphite-containing regions in the cast iron. Therefore, before corrosion, the two surface treatments changed the initial surface state from direct cast-iron exposure to a barrier-controlled condition in which surface-region continuity, thickness uniformity, and defects can strongly affect corrosion initiation.

### 3.2. Quantitative Corrosion Behavior

**Figure 5:**
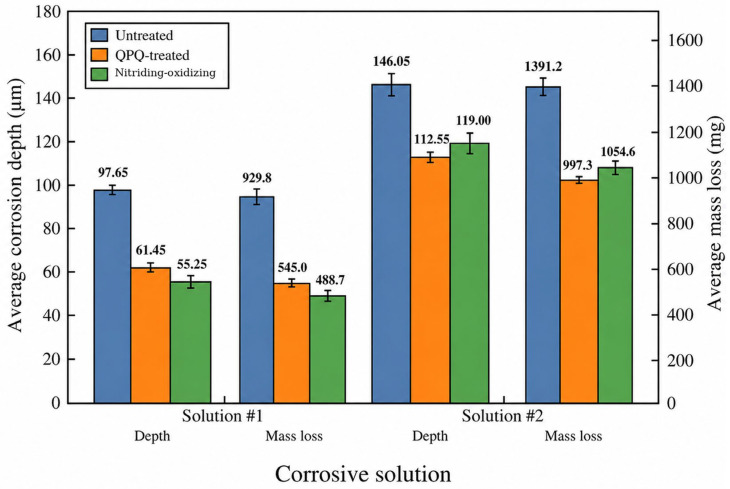
Statistical bar chart comparing the mass-loss-equivalent average corrosion depth and average mass loss of untreated, QPQ-treated, and nitriding–oxidizing-treated specimens after immersion in Solution #1 (5 vol% formic acid–NaCl, 60 °C) and Solution #2 (10 vol% formic acid–NaCl, 80 °C). Error bars represent standard deviations from two parallel specimens.

Figure 5 summarizes the mass-loss-equivalent average corrosion depth and average mass loss of the untreated, QPQ-treated, and nitriding–oxidizing-treated specimens after immersion in Solution #1 #2. Both indices follow the same overall trend. When the corrosive medium changed from Solution #1 to Solution #2, both corrosion depth and mass loss increased for all surface states, indicating that the higher formic acid concentration and elevated temperature accelerated material dissolution and corrosion penetration in the formic acid–NaCl medium.

For the untreated substrate, the average corrosion depth increased from 97.65 μm in Solution #1 to 146.05 μm in Solution #2, and the average mass loss increased from 929.8 mg to 1391.2 mg. The corresponding increases were 49.57% and 49.62%, respectively. The close agreement between the depth increase and mass-loss increase indicates that the mass-loss-equivalent depth reasonably reflected the global material removal under the two accelerated corrosion conditions.

The two surface-modified specimens exhibited lower corrosion depth and mass loss than the untreated substrate under both solution conditions. In Solution #1, the QPQ-treated specimen showed an average corrosion depth of 61.45 μm and an average mass loss of 545.0 mg, corresponding to reductions of 37.07% and 41.39% relative to the untreated substrate. The nitriding–oxidizing-treated specimen showed an average corrosion depth of 55.25 μm and an average mass loss of 488.7 mg, corresponding to reductions of 43.42% and 47.44%, respectively. These results show that both treatments provided initial protection in the lower-severity medium, with the nitriding–oxidizing-treated specimen showing a slightly stronger protective effect under this condition.

In Solution #2, the protective advantage of the modified surface regions decreased. The QPQ-treated specimen exhibited an average corrosion depth of 112.55 μm and an average mass loss of 997.3 mg, which were 22.94% and 28.31% lower than those of the untreated substrate. The nitriding–oxidizing-treated specimen exhibited an average corrosion depth of 119.00 μm and an average mass loss of 1054.6 mg, corresponding to reductions of 18.52% and 24.19%, respectively. Although both surface treatments still reduced corrosion, the difference between treated and untreated specimens became smaller under the more aggressive condition.

The increase from Solution #1 to Solution #2 was more pronounced for the surface-modified specimens than for the untreated substrate. For the QPQ-treated specimen, corrosion depth and mass loss increased by 83.16% and 82.99%, respectively. For the nitriding–oxidizing-treated specimen, the corresponding increases reached 115.38% and 115.80%. This indicates that the modified surface regions provided initial protection but became more vulnerable under the more aggressive medium once local penetration or surface-region discontinuity occurred. Therefore, cross-sectional SEM-BSE morphology and selected EDS elemental mapping were used to assess whether corrosion proceeded mainly by uniform dissolution or by localized penetration through weak regions.

### 3.3. Cross-Sectional Morphology, EDS Mapping, and Corrosion Mechanism

**Figure 6:**
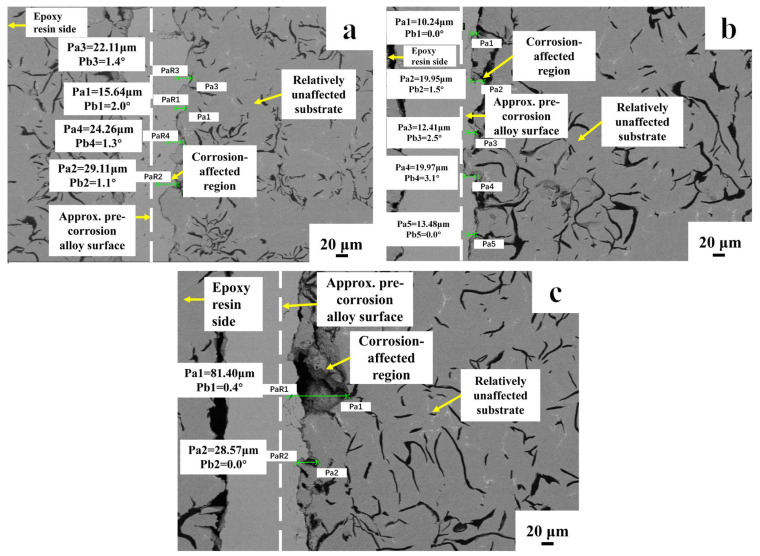
Cross-sectional SEM-BSE images of specimens after immersion in Solution #1 (5 vol% formic acid–NaCl, 60 °C): (**a**) untreated substrate; (**b**) QPQ-treated specimen; and (**c**) nitriding–oxidizing-treated specimen. The epoxy-resin side, approximate pre-corrosion alloy surface, corrosion-affected region, and relatively unaffected substrate are marked in each local cross-sectional image. The dashed lines indicate the approximate pre-corrosion alloy surface. The yellow arrows indicate the annotated regions in the images, and the green double-headed arrows/dimension annotations indicate local corrosion-affected widths measured at selected positions.

**Figure 7:**
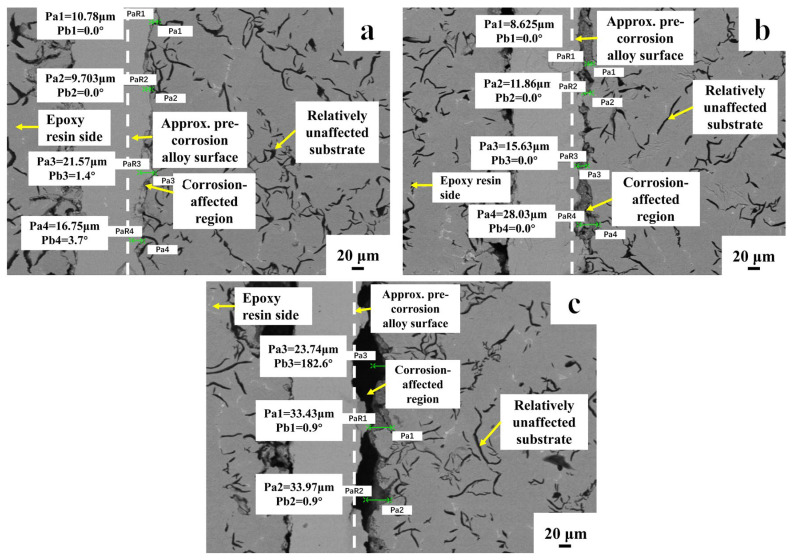
Cross-sectional SEM-BSE images of specimens after immersion in Solution #2 (10 vol% formic acid–NaCl, 80 °C): (**a**) untreated substrate; (**b**) QPQ-treated specimen; and (**c**) nitriding–oxidizing-treated specimen. The epoxy-resin side, approximate pre-corrosion alloy surface, corrosion-affected region, and relatively unaffected substrate are marked in each local cross-sectional image. The dashed lines indicate the approximate pre-corrosion alloy surface. The yellow arrows indicate the annotated regions in the images, and the green double-headed arrows/dimension annotations indicate local corrosion-affected widths measured at selected positions.

To relate the quantitative corrosion behavior to microscopic evidence, the corrosion mechanism was interpreted based on cross-sectional SEM-BSE morphology together with selected EDS elemental mapping. The SEM-BSE images were used to identify the epoxy-resin side, approximate pre-corrosion alloy surface, corrosion-affected region, relatively unaffected substrate, and local corrosion-front morphology. The selected EDS maps were used to correlate the distributions of C, O, and Fe with these marked morphological regions. Therefore, the mechanism analysis was based on combined morphological and elemental-distribution evidence, while EDS mapping was not used alone for direct phase identification. Figure 6 and Figure 7 show representative cross-sectional SEM-BSE morphologies after immersion in Solution #1 #2, respectively. The marked dimensions represent local corrosion-affected widths at selected positions. Because corrosion in graphite-containing cast iron is highly nonuniform, these local values are used to describe representative corrosion-front morphology and local penetration features rather than to replace the mass-loss-equivalent average corrosion depths in Figure 5.

After immersion in Solution #1, the untreated substrate exhibited a discontinuous corrosion front near the exposed edge (Figure 6a). The local affected widths in the selected field were approximately 15.64–29.11 μm, and the damaged boundary developed grooves rather than a flat dissolution front. The stripe-like contrast near the interface should not be regarded as an independent continuous layer with a definite composition. It corresponds to a locally corrosion-affected boundary formed near the approximate pre-corrosion alloy surface, where local dissolution, corrosion damage, and SEM-BSE contrast changes overlap. The epoxy-resin side and approximate pre-corrosion alloy surface are indicated to define the cross-sectional orientation of the local high-magnification field. These features indicate preferential attack at structurally heterogeneous regions of the high-alloy bainitic gray cast iron. Recent studies on gray cast iron exposed to marine environments also reported that corrosion morphology and corrosion products are strongly affected by graphite-containing microstructures and local microstructural heterogeneity [27,28]. Thus, the untreated substrate should be interpreted as a heterogeneous corrosion system rather than a uniformly dissolving metallic surface.

The QPQ-treated specimen showed a more restricted corrosion-affected region under Solution #1 (Figure 6b). Most marked widths were approximately 10.24–19.97 μm, generally smaller than those in the untreated substrate. This result indicates that the nitrocarburized/oxidized surface region delayed the initial transport of the formic acid–NaCl medium toward the substrate. For the nitriding–oxidizing-treated specimen (Figure 6c), one selected position showed a corrosion-affected width of approximately 81.40 μm, whereas another position was approximately 28.57 μm. The larger local value reflects a defect-sensitive penetration site in the observed field and should not be interpreted as the average corrosion depth of the whole specimen. The regions marked as corrosion-affected regions in Figure 6b,c correspond to damaged zones rather than independent, compositionally defined layers. Similar defect-sensitive protection of nitrided or oxidized surfaces has been reported in aggressive halide-containing environments [32,38].

The chemical driving force in the lower-severity medium is associated with weak-acid dissociation and local surface heterogeneity. Formic acid dissociates near the metal/solution interface according to

(2) $$HCOOH\;⇌\;H^{+}+{HCOO}^{-}$$

At exposed iron-rich regions, anodic dissolution and cathodic hydrogen evolution can be expressed as

(3) $$Fe\;\to \;{Fe}^{2+}\;+\;2e^{-}$$

(4) $$2H^{+}+2e^{-}\;\to \;H_{2}↑$$

The dissolved *Fe*^2+^ may further interact with *HCOO*^−^ to form soluble iron-formate species:

(5) $${Fe}^{2+}\;+\;2{HCOO}^{-}\;⇌\;{Fe(HCOO)}_{2}(aq)$$

A simplified overall reaction for acid-assisted dissolution can therefore be expressed as

(6) $$Fe+2HCOOH\;\to \;{Fe(HCOO)}_{2}(aq)+H_{2}↑\;$$

Sulejmanovic et al. [16] reported that formic acid and related carboxylic acids play an important role in steel corrosion and may be associated with iron-formate species or corrosion products. Eslami et al. [17] further reported that HCOOH can reduce the protectiveness of magnetite-type surface layers at elevated temperatures. These findings support the present interpretation that formic acid accelerates corrosion not only by supplying H^+^ but also by destabilizing compact corrosion products through formate-related complexation.

When the corrosive condition changed to Solution #2, the cross-sectional morphology became rougher and more irregular (Figure 7). Although the marked widths in one selected field of view cannot be directly compared with the mass-loss-equivalent average depths, the SEM-BSE images show that the corrosion boundary became less uniform and the damaged regions became more continuous along the exposed edge. In Figure 7, the marked corrosion-affected regions correspond to locally damaged regions formed inward from the approximate pre-corrosion alloy surface, whereas the regions on the matrix side retain the typical graphite-containing cast-iron microstructure and are therefore marked as relatively unaffected substrate. This morphological trend is consistent with Figure 5, where all specimens exhibited higher corrosion depth and mass loss in Solution #2.

For the untreated substrate in Solution #2 (Figure 7a), the selected local widths were approximately 9.70–21.57 μm. These values represent local positions in a highly nonuniform corrosion front and should not be compared one-to-one with the selected widths in Figure 6a or with the mass-loss-equivalent average corrosion depth. Nevertheless, the morphology shows a more continuous damaged edge and greater interfacial roughness, indicating that the higher formic acid concentration and elevated temperature increased the probability of localized dissolution events. For the QPQ-treated specimen (Figure 7b), several local regions with widths of approximately 8.63–28.03 μm were observed along the modified surface region. The corrosion front remained less extensive than in the untreated substrate but became more uneven, indicating that the QPQ-treated surface still provided partial protection while its barrier function weakened as acidity and temperature increased. For the nitriding–oxidizing-treated specimen (Figure 7c), the local affected widths were approximately 23.74–33.97 μm, and localized damage became more pronounced. This indicates that the oxygen-enriched outer region and N-enriched nitrided region delayed direct exposure during the initial stage, but local discontinuities became penetration channels under the more aggressive condition.

Chloride ions may intensify local penetration by promoting film breakdown and sustaining local acidification in damaged regions. The simplified chloride-assisted reactions can be written as

(7) $${Fe}^{2+}\;+\;{Cl}^{-}\;⇌\;{FeCl}^{+}(aq)\;$$

(8) $${FeCl}^{+}+H_{2}O\;⇌\;{FeOH}^{+}+H^{+}+{Cl}^{-}\;$$

These reactions illustrate how *Cl*^−^ may participate in soluble intermediate species and be regenerated during hydrolysis, which favors localized acidification and continued dissolution at weak positions. In this work, the role of chloride is interpreted from the NaCl-containing corrosive medium and the established chloride-assisted depassivation/film-breakdown mechanism, rather than from direct identification of chloride-containing corrosion products by EDS mapping. Therefore, the higher acid concentration, elevated temperature, and chloride-containing environment jointly promote inward penetration once the modified surface region is locally interrupted.

**Figure 8:**
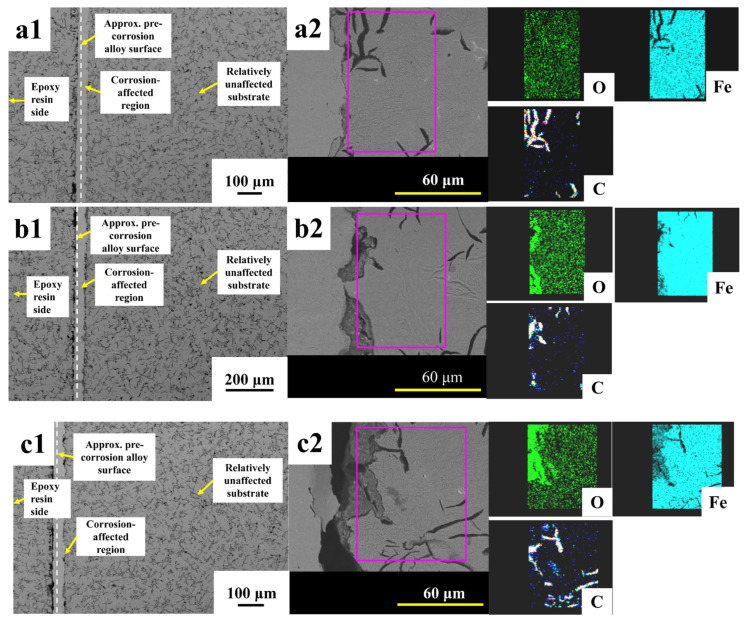
Overview SEM-BSE images, local SEM-BSE images, and selected EDS elemental mapping results of specimens after corrosion in Solution #2 (10 vol% formic acid–NaCl, 80 °C): (**a1**,**a2**) untreated substrate; (**b1**,**b2**) QPQ-treated specimen; and (**c1**,**c2**) nitriding–oxidizing-treated specimen. The overview SEM-BSE images show the epoxy-resin side, approximate pre-corrosion alloy surface, corrosion-affected region, and relatively unaffected substrate. The purple rectangles in the local SEM-BSE images indicate the regions selected for EDS elemental mapping. The mapping images show the selected elemental distributions of C, O, and Fe.

Figure 8 combines cross-sectional SEM-BSE morphology and selected EDS elemental mapping to support the mechanism interpretation. The figure should be read in two steps. First, the overview SEM-BSE images define the cross-sectional orientation, including the epoxy-resin side, approximate pre-corrosion alloy surface, corrosion-affected region, and relatively unaffected substrate. Second, the local SEM-BSE images show the exact regions selected for EDS elemental mapping, as marked by the purple rectangles. The C, O, and Fe maps are interpreted within these marked regions and correlated with the SEM-BSE morphology. In the untreated substrate (Figure 8(a1,a2)), the C map mainly corresponds to flake graphite in the gray cast iron matrix, whereas the Fe map identifies the metallic substrate and the interruption of the matrix near the damaged boundary. A relatively higher O signal is observed near the outer corrosion-affected region, suggesting the possible presence of oxygen-containing corrosion products or oxidized regions. The O map is interpreted together with SEM-BSE morphology because the O signal alone cannot determine the exact oxide or corrosion-product phase. Therefore, the EDS maps are used as correlative elemental-distribution evidence and are not used alone for direct phase identification.

For the QPQ-treated specimen (Figure 8(b1,b2)), the selected O map shows a relatively higher signal near the surface-side damaged region, and the Fe map remains mainly associated with the matrix side. The degradation of the QPQ-treated surface is assessed from SEM-BSE morphology, the local corrosion-affected widths in Figure 7b, and the pre-corrosion surface-region characterization. Ni et al. [39] reported that QPQ-treated steels can improve corrosion resistance through a multilayer surface structure, but porosity or spallation may reduce protection. The present results therefore support a mechanism in which surface-region discontinuities become pathways for acid- and chloride-assisted penetration.

For the nitriding–oxidizing-treated specimen (Figure 8(c1,c2)), the O map shows a relatively higher signal near the outer damaged region, while the Fe map indicates the metallic matrix and the damaged boundary. EDS mapping cannot distinguish adsorbed Cl^−^, soluble Fe–Cl complexes, residual salt or solution trapped in pores, or chloride associated with corrosion products; therefore, no specific chloride-containing species are assigned from EDS mapping. The local damage shown in Figure 7c is interpreted as the result of acid-assisted dissolution, chloride-assisted film breakdown, and penetration through local discontinuities. Possible oxygen-containing corrosion reactions can be schematically represented by hydrolysis and oxidation reactions such as

(9) $${Fe}^{2+}\;+\;2H_{2}O\;⇌\;{Fe(OH)}_{2}+2H^{+}\;$$

(10) $${4Fe(OH)}_{2}+O_{2}\;\to \;2{Fe}_{2}O_{3}+4H_{2}O\;$$

Equations (9) and (10) illustrate possible oxygen-containing corrosion reactions and are not used as direct phase-identification evidence. The C/O/Fe EDS maps provide elemental-distribution support for the marked SEM-BSE morphological regions, while the interpretation of corrosion-product formation and modified-surface-region degradation is based on the combined SEM-BSE morphology, selected elemental distributions, and corrosion mechanism analysis.

To further support the qualitative EDS mapping results and address the oxygen-related quantification, a semi-quantitative image-based analysis was performed on the O Kα EDS maps of the corroded samples. The same RGB-thresholding criterion was applied to all maps, with the black background outside the effective mapping region and the image labels excluded from the calculation. The O-enriched area fraction was calculated according to Equation (11):

(11) $$A_{O}\;=\;\frac{N_{O}}{N_{total}}\;\times \;100\%\;$$

where *A_O_* is the oxygen-enriched area fraction, *N_O_* is the number of oxygen-enriched pixels identified using the same RGB-thresholding criterion, and *N_total_* is the total number of pixels in the effective O Kα mapping area. The obtained values represent relative oxygen-enriched area fractions rather than absolute oxygen concentration in wt% or at.%.

The selected O maps in Figure 8 show that oxygen signals are mainly distributed near the corrosion-affected surface regions and modified surface regions. However, the color intensity in EDS maps provides only a qualitative comparison and cannot by itself distinguish whether the oxygen signal originates from corrosion products, residual oxide-containing modified regions, or their coexistence. Therefore, the calculated O-enriched area fraction was used as a relative indicator to compare the spatial extent of oxygen-enriched regions under the same image-processing criterion. For the untreated substrate, an increase in this fraction under Solution #2 would indicate enhanced formation and accumulation of oxygen-containing corrosion products. For the QPQ-treated and gas nitriding–oxidizing-treated samples, the value reflects the combined contribution of oxygen-containing corrosion products and the oxygen-bearing modified surface region; therefore, it should not be directly interpreted as corrosion severity alone. The O maps used for the semi-quantitative calculation included all corroded samples under Solution #1 #2, while representative maps are shown in Figure 8. The semi-quantitative results are summarized in Table 4.

The slightly lower O-enriched area fraction of the gas nitriding–oxidizing-treated sample in Solution #2 compared to Solution #1 does not indicate reduced corrosion severity. Instead, it may be related to the local detachment, redistribution, or discontinuous preservation of the oxygen-containing modified layer and corrosion products in the selected mapping region. Therefore, this parameter should be interpreted together with mass-loss results and cross-sectional SEM-BSE morphology.

Accordingly, these semi-quantitative values provide numerical support for the O-map observations and are used together with the SEM-BSE morphology and elemental distributions to interpret the corrosion mechanism.

Based on the cross-sectional SEM-BSE morphology in Figure 6 and Figure 7, the selected C/O/Fe elemental distributions in Figure 8, and the semi-quantitative O-enriched area fractions in Table 4, the corrosion process can be interpreted as a surface-region- and defect-controlled penetration mechanism. For the untreated substrate, SEM-BSE images show direct exposure of the graphite-containing bainitic matrix and an irregular corrosion-affected boundary, while the C map corresponds mainly to flake graphite and the Fe map shows matrix interruption near the damaged region. These features indicate localized dissolution along graphite/matrix interfaces and other microstructurally heterogeneous regions. For the QPQ-treated specimen, the modified surface region reduced direct electrolyte access, but SEM-BSE morphology together with the O/Fe maps shows that corrosion preferentially developed at discontinuities or weakly protected surface-side regions. For the gas nitriding–oxidizing-treated specimen, the oxygen-enriched outer region and N-enriched nitrided region formed a gradient barrier before corrosion; however, SEM-BSE morphology and selected EDS mapping indicate local penetration where this barrier became discontinuous or chemically weakened. Therefore, the reduced corrosion resistance under Solution #2 was mainly associated with localized penetration through graphite-containing regions and discontinuous modified surface regions, rather than uniform thinning alone. The degradation mechanism can be summarized as follows: formic acid promotes dissolution of exposed Fe-rich matrix regions and formate-related complexation; graphite and the bainitic matrix create local electrochemical heterogeneity; QPQ and gas nitriding–oxidizing treatments initially reduce direct electrolyte access to graphite/matrix interfaces and modified surface regions, but local defects and discontinuities provide penetration paths; and Cl^−^ in the NaCl-containing medium accelerates film breakdown and localized corrosion once the surface region is damaged.

## 4. Conclusions

Under the accelerated laboratory immersion conditions used in this study, high-alloy bainitic gray cast iron cylinder-liner specimens with untreated, QPQ-treated, and nitriding–oxidizing-treated surfaces all exhibited increased mass-loss-equivalent average corrosion depth, mass loss, and annualized corrosion-rate index when the formic acid–NaCl environment changed from the lower-severity condition to the more aggressive condition. For the untreated substrate, the mass-loss-equivalent average corrosion depth increased from 97.65 ± 1.77 to 146.05 ± 5.02 μm, whereas the mass loss increased from 929.8 ± 22.1 to 1391.2 ± 64.3 mg. For the QPQ-treated specimens, the mass-loss-equivalent average corrosion depth increased from 61.45 ± 1.34 to 112.55 ± 1.91 μm, and the mass loss increased from 545.0 ± 6.3 to 997.3 ± 8.6 mg. For the nitriding–oxidizing-treated specimens, the mass-loss-equivalent average corrosion depth increased from 55.25 ± 2.33 to 119.00 ± 4.67 μm, whereas the mass loss increased from 488.7 ± 29.1 to 1054.6 ± 34.2 mg. The corresponding annualized corrosion-rate indices increased from 855.41 ± 15.49 to 1279.40 ± 43.98 mm year^−1^ for the untreated substrate, from 538.30 ± 11.77 to 985.94 ± 16.72 mm year^−1^ for the QPQ-treated specimens, and from 483.99 ± 20.44 to 1042.44 ± 40.88 mm year^−1^ for the nitriding–oxidizing-treated specimens. The consistent increase in these quantitative parameters demonstrates that the more aggressive formic acid–NaCl condition accelerated corrosion in all surface states.

Under the lower-severity formic acid–NaCl condition, both surface treatments reduced corrosion compared with the untreated substrate. The nitriding–oxidizing treatment produced the lowest mass-loss-equivalent average corrosion depth, whereas the QPQ-treated specimens also showed significant improvement. The enhanced protection was associated with the formation of modified surface regions, including a nitrocarburized/oxidized surface region in the QPQ-treated specimens and an oxygen-enriched outer region combined with an N-enriched nitrided region in the nitriding–oxidizing-treated specimens. These regions reduced direct electrolyte access to graphite/matrix interfaces and thereby reduced the opportunity for graphite–matrix microgalvanic corrosion during the initial exposure stage.

The untreated substrate corroded preferentially near graphite–matrix interfaces because flake graphite and the bainitic matrix formed electrochemically heterogeneous regions. In formic acid solution, H^+^ sustained cathodic hydrogen evolution, whereas HCOO^−^ could complex with Fe^2+^ and hinder the formation of compact protective corrosion products. This combined effect promoted porous corrosion layers, matrix undercutting, and corrosion penetration along the graphite network, explaining the greater vulnerability of the untreated material under aggressive conditions.

The QPQ and nitriding–oxidizing treatments functioned as initial corrosion barriers; however, their degradation under aggressive formic acid–NaCl conditions was governed primarily by localized defects and discontinuities rather than by uniform dissolution of the modified surface regions. Acid attack promoted localized degradation at weak regions within the modified surface regions, formate complexation maintained the exposed matrix in an active dissolution state, and graphite–matrix microgalvanic corrosion, together with capillary penetration along graphite interfaces, drove the corrosion front inward. These findings provide a practical basis for the design of low-cost, corrosion-resistant surface treatments for methanol-engine cylinder liners, particularly with respect to the compactness of the modified surface regions, defect control, and graphite-interface protection. The semi-quantitative O Kα map analysis further supports the oxygen-related mechanism by showing increased O-enriched area fractions under the more aggressive condition for the untreated and QPQ-treated samples, while the high O-enriched area fraction of nitriding–oxidizing-treated samples reflects both the oxide-containing modified layer and corrosion products.

## Figures and Tables

**Table 1:** Chemical composition of high-alloy bainitic gray cast iron (wt%).

Element	Symbol	Controlled Range (wt%)
Carbon	C	2.6–3.2
Silicon	Si	1.7–2.5
Nickel	Ni	0.8–1.5
Molybdenum	Mo	1.0–1.8
Sulfur	S	<0.10
Manganese	Mn	0.4–1.0
Copper	Cu	0.2–0.8
Chromium	Cr	≤0.3

**Table 2:** Processing parameters of QPQ and gas nitriding–oxidizing treatments.

Treatment	Step	Process	Atmosphere/Medium	Temperature	Time	Cooling
QPQ treatment	1	Preheating	Furnace preheating	400 °C	1 h	—
QPQ treatment	2	Salt-bath nitrocarburizing	Salt bath	540 °C	3.5 h	—
QPQ treatment	3	Oxidizing	Oxidizing medium	390 °C	20 min	—
QPQ treatment	4	Cooling	Water	—	—	Water cooling
Gas nitriding–oxidizing treatment	1	N_2_ atmosphere preheating	N_2_	350 °C	1 h	—
Gas nitriding–oxidizing treatment	2	Gas nitriding	NH_3_	540 °C	4 h	—
Gas nitriding–oxidizing treatment	3	Steam oxidizing	H_2_O/steam	415 °C	1 h	—
Gas nitriding–oxidizing treatment	4	Cooling	Furnace	—	—	Furnace cooling

**Table 3:** Composition and test conditions of formic acid–NaCl corrosive solutions.

Corrosive Solution No.	Formic Acid Addition	NaCl Addition	Final Volume	Nominal Formic Acid Volume Fraction	Test Temperature	Immersion Time
#1	50 mL	2.5 g	1000 mL	5 vol%	60 °C	60 min
#2	100 mL	2.5 g	1000 mL	10 vol%	80 °C	60 min

**Table 4:** Semi-quantitative image-based analysis of oxygen-enriched regions from O Kα EDS maps.

Sample	Corrosion Condition	Magnification	O-Enriched Area Fraction/%
Untreated substrate	Solution #1 (5 vol% formic acid–NaCl, 60 °C)	2000×	37.5
Untreated substrate	Solution #2 (10 vol% formic acid–NaCl, 80 °C)	2000×	56.1
QPQ-treated sample	Solution #1 (5 vol% formic acid–NaCl, 60 °C)	2000×	61.2
QPQ-treated sample	Solution #2 (10 vol% formic acid–NaCl, 80 °C)	2000×	70.6
Gas nitriding–oxidizing-treated sample	Solution #1 (5 vol% formic acid–NaCl, 60 °C)	2000×	69.4
Gas nitriding–oxidizing-treated sample	Solution #2 (10 vol% formic acid–NaCl, 80 °C)	2000×	64.7

Note: The O-enriched area fraction was obtained from the O Kα EDS maps by semi-quantitative image-based analysis using the same RGB-thresholding criterion for all images. The black background outside the effective mapping region and image labels were excluded from the calculation. The values represent the relative area fraction of oxygen-enriched pixels in the analyzed EDS maps, rather than the absolute oxygen concentration in wt.% or at.%.

## Data Availability

The original contributions presented in this study are included in the article. Further inquiries can be directed to the corresponding authors.
